# Aquaporins in Health and Disease: An Overview Focusing on the Gut of Different Species

**DOI:** 10.3390/ijms17081213

**Published:** 2016-07-27

**Authors:** Alessandra Pelagalli, Caterina Squillacioti, Nicola Mirabella, Rosaria Meli

**Affiliations:** 1 Department of Advanced Biomedical Sciences, University of Naples “Federico II”, Via Pansini 5, 80131 Naples, Italy; 2 Institute of Biostructures and Bioimages, National Research Council, Via De Amicis 95, 80131 Naples, Italy; 3 Department of Veterinary Medicine and Animal Productions, University of Naples “Federico II”, Via Veterinaria 1, 80137 Naples, Italy; caterina.squillacioti@unina.it (C.S.); nicola.mirabella@unina.it (N.M.); 4 Department of Pharmacy, University of Naples “Federico II”, Via D. Montesano 49, 80131 Naples, Italy; rosaria.meli@unina.it

**Keywords:** aquaporins, gut, physiology, pathology, human and animal species

## Abstract

Aquaporins (AQPs) play a pivotal role in gut homeostasis since their distribution and function is modulated both in physiological and in pathophysiological conditions. The transport of water and solutes through gut epithelia is essential for osmoregulation and digestive and absorptive functions. This passage is regulated by different AQP isoforms and characterized by their peculiar distribution in the gastrointestinal tract. To date, AQP localization has been identified in the gut and associated organs of several mammalian species by different techniques (immunohistochemical, western blotting, and RT-PCR). The present review describes the modulation of AQP expression, distribution, and function in gut pathophysiology. At the same time, the comparative description of AQP in animal species sheds light on the full range of AQP functions and the screening of their activity as transport modulators, diagnostic biomarkers, and drug targets. Moreover, the phenotype of knockout mice for several AQPs and their compensatory role and the use of specific AQP inhibitors have been also reviewed. The reported data could be useful to design future research in both basic and clinical fields.

## 1. Introduction

Since the discovery of aquaporins (AQPs) by Peter Agre in 1992 [1], research in this channel protein field has continuously evolved, resulting in enhanced knowledge of their physiopathological role. Aquaporins show a wide distribution both in different organisms (bacteria, plants, and animals) and tissues, and a selective activity in conducting water molecules in and out of cells and preventing the passage of ions and other solutes [2]. Functionally AQPs are divided into three subfamilies: (a) orthodox AQPs (AQP1, 2, 4, and 5), which are selectively permeable for water; (b) aquaglyceroporins (AQP3, 7, 9, and 10), which are permeable to water as well as to glycerol, urea, and/or other small solutes; and (c) unorthodox aquaporins (AQP6, 8, 11, and 12), with peculiar intracellular localization [3,4] and functions [5,6,7]. Thirteen proteins have been identified, differing in size from 27 kDa (AQP8) to 37 kDa (AQP7), with diverse water permeabilities [8,9].

During the last two decades their role in gut physiology as proteins regulating multiple processes including the transfer of water as well as ions, solutes, and nutrients and feces constitution has attracted particular attention [10]. The study of the specific characteristics of aquaporin vs. glyceroporins is based on their different peculiarities, such as pore gating. This has provided new insights into structure–function relationships, as well as mechanisms of regulation, and into their diverse physiologic roles (for a review, see [11]).

AQP organization, demonstrated by crystallographic studies as well, allows these channels to be defined as small intrinsic proteins with a specific permeability established by pore diameter due to the assembly of the different constituents (domains) [12]. According to this concept, AQPs play a major role in gut pathophysiology in the new clinical and therapeutic approaches to several diseases [13].

Large quantities of water molecules may transfer along the epithelia by different pathways (paracellular, transcellular, or both) according to the osmotic gradient resulting from the passive passage of ions and solutes [14]. Water transport occurs from the blood to the lumen or in the opposite direction, allowing other mechanisms related to it, i.e., secretion of hormones or factor release. Moreover, water transfer in gut lumen and its removal, such as feces formation, define a different role and compartmentalization of aquaporins throughout the entire intestinal tract [15].

Here we review the role of AQPs in the gut, focusing on their specific involvement and modification both in physiological and in pathological conditions, with particular reference to the differences between humans and animals.

## 2. AQP Structure and Distribution in Gut

The coordinate passage of molecules and the absorption and secretion of electrolyte and fluids across the intestinal epithelium are important processes for gut homeostasis [16]. In fact, the gut represents an intelligent sensitive organ in continuous communication with the external environment with several regulatory activities due to different specialized immune cells and factors (i.e., nutrients, hormones, microbiota). To date, several intestinal AQPs have been identified [17], although their tissue characterization has not yet been completely defined. Both crystallographic, for the definition of structural features, and immunohistochemical studies have been performed. According to the particular organization of the intestinal tract (small intestine, large intestine, and liver), its morpho-anatomy and its different functions, specific AQPs have been located and water transcellular regulatory activities identified. Notably, AQPs were found in red blood cells for the first time in 1986, and subsequently they were identified as AQP1 [18].

**Figure 1:**
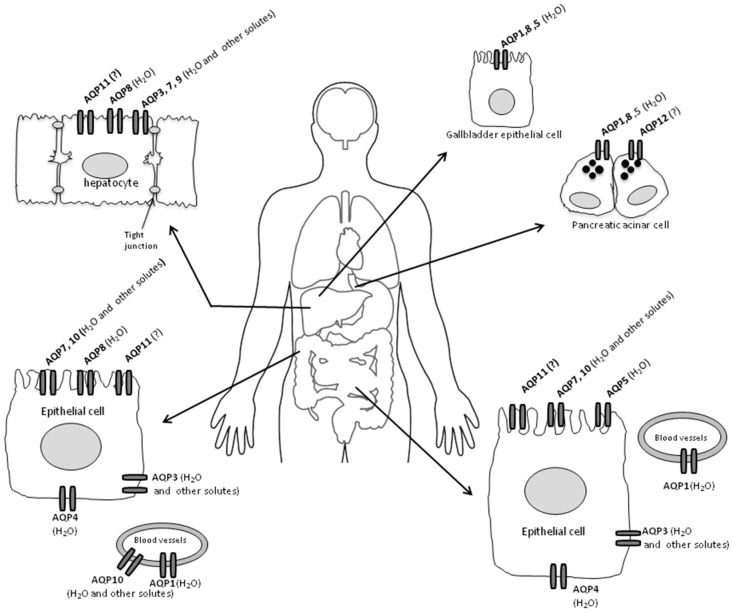
Main AQPs’ cell polarity and channel protein distribution in the gastrointestinal tract and associated organs. (?) = unknown role or function. For the specific intracellular localization of different AQPs see text.

In Table 1 the gut distribution of several AQPs, their particular selective permeability, and cellular localization are summarized and related citations reported. Moreover, in Figure 1 AQP cell polarity is shown, reporting channel protein distribution in the gastrointestinal tract and associated organs.

### 2.1. Small Intestine

The small intestine along its specific tracts (duodenum, jejunum, and ileum) has been intensively investigated, showing the expression of at least nine AQPs (AQP1, 2, 3, 5, 7, 8, 9, 10, and 11) [21,41]. It is well known that the proximal segments of intestine are characterized by osmotic permeability and by secretive activities. In particular, at the duodenum level AQ1, 3, 7, 10, and 11 are mainly expressed.

Among these, AQP1 is the best-known and most widely expressed along the capillary endothelium of the ileum mucosa and submucosa [20]. A structural molecular study on AQP1 showed that 50% of the protein is expressed as a glycosylated form by a 5.4 kDa polylactosaminyl oligosaccharide at residue N42 in the first extracellular loop [42]. Immunohistochemical studies evidenced both AQP2 and AQP3 distribution in the entire small intestine, albeit with a more limited tissue expression of AQP2 than of AQP3 [25]. In fact, AQP2 represents one of the less-studied proteins since only one paper reported its expression at the intestinal level, probably indicating its marginal role in this tissue [25]. AQP5 was defined by Parvin et al. [30] as an exocrine-type water channel for its granule secretory activity. It has been well characterized by immunohistochemical and western blotting alongside the rat duodenum, together with AQP1, showing a classic protein profile characterized by a band at 27 kDa (AQP1) and another at 28 kDa (AQP5), and their glycosylated forms (35- to 50-kDa).

Research into AQP7 and AQP8 immunolabeling and PCR studies demonstrated their protein expression on epithelial cells of the rat small intestine [43]. Similarly, RT-PCR studies revealed AQP8 mRNA expression not only in the rat jejunum, but also in liver hepatocytes and pancreas acinar cells, demonstrating a peculiar distribution of this AQP [44]. Other data from Elkjær et al. [45] indicate that AQP8 is also distributed in intracellular compartments, suggesting its role in osmotic function between cytoplasma and vescicular compartments in several tissues including small intestine. By contrast, the AQP9 expression has been evidenced by multiple analytical techniques (immunohistochemical, RT-PCR, and western blotting) particularly along the epithelial cells in the ileum and duodenum region [26,35], probably in goblet cells.

Moreover, AQP10 has been discovered and identified in the duodenum, jejunum, and ileum of humans [36,37,46]. The authors suggested that water passes the apical membrane of the epithelia principally through AQP10 and partly through AQP8, and the basolateral membrane through AQP3 [46]. Conversely, genetic studies demonstrated that the mouse AQP10 gene contains several mutations that lead to proteins without functional activity [38]. Such data were supported by knockout mice studies leading to the determination of a minimum role of some AQPs in gastro-intestinal (GI) tracts [2,47]. Thus, the absence of AQP10 in mice suggests that this AQP could play a special role in glycerol absorption in humans [47,48].

Recently, AQP11 has been discovered and well characterized in the human brain, showing a lower similarity to other well-known mammalian AQPs and aquaglyceroporins [49]. This protein has been described in the human duodenum [21,31].

### 2.2. Large Intestine

With respect to the small intestine, the distribution of AQPs in the large intestine also mirrors their involvement in the different processes set up herein. In the large intestine six AQPs have been identified (AQP1, 2, 3, 4, 7, and 8), not always distributed evenly in the same tracts (cecum, colon, and rectum) [21,41]. Their presence has been demonstrated by RT-PCR and immunohistochemical study [45,48]. A study by Laforenza et al. [21] showed that large quantities of AQP1 were expressed in colonic mucosa.

Other authors demonstrated that AQP2 is present in the rat distal colon and that its role can be modulated by vasopressin [50]. Furthermore, AQP3 localization has been observed in the large intestine at the level of the stratified and basolateral epithelia in the distal colon [26,28,29,51] and in the rectum, whereas no signal detection was found in the caecum [52]. AQP4 has also been found in humans, albeit with a very low immunoreactive signal [21]. Moreover, functional studies on AQP4-knockout mice demonstrated that AQP4 deletion resulted in the reduction of water permeability in the proximal but not the distal colon, showing its role in transcellular water movement across surface colonocytes. Interestingly, colonocytes play little or no role in fecal dehydration and colonic fluid secretion [53].

AQP7 and AQP8 were also demonstrated to be expressed in the cecum, proximal and distal colon and rectum, albeit with a reduced intensity compared with the small intestine [54]. In particular, studies on the colons of AQP8-knockout mouse revealed their involvement in the regulation of different enzymes implicated in carbohydrate metabolism, using semi-quantitative, fluorescence-stained, two-dimensional gel electrophoresis (2-DE) coupled with nano LC-Ms/Ms [55].

### 2.3. Liver

Given the close organ association between the intestine and the liver, created by bile, hormones, inflammatory mediators, and products of digestion and absorption [56], the contribution of AQPs in their functions must be considered. In particular, the liver strongly expresses at least six AQPs (AQP1, 3, 7, 8, 9, and 11) [22,39,41,57]. Immunohistochemical studies revealed the expression of AQPs in different hepatic cell types, such as in cholangiocytes (AQP1 and AQP7), endothelial cells (AQP1), Kupffer cells (AQP3), and hepatocytes, (AQP7, 8, and 9) [22].

Recently, Ishibashi et al. [39] reviewed mammalian superaquaporins (AQP11 and AQP12), focusing on their roles, which are only speculated by the phenotypes of their null mutants. For example, the water transport through the superaquaporin inside the cell will be important for the cell organelle function; the facilitated vesicle-to-plasma membrane fusion will be controlled by water transport through vesicular AQPs, as suggested by AQP12 null mice [40]. AQP11 is also expressed in the liver and its knockout produced intracellular vacuoles in the hepatocyte around the portal area, which was more pronounced by fasting in the liver of AQP11 knockout mice [58].

### 2.4. Pancreas and Gallbladder

In recent years, the involvement of AQPs also in the pancreas and gallbladder has been investigated in relationship to their role in the secretion and reabsorption of water in pancreatic juice and in the formation of gall bladder stones, respectively. As shown in Table 1, different AQPs have been also identified in the pancreas (AQP1, 5, 8, and 12) [23,33]. Immunohistochemical and functional studies have partially clarified their physiological role. In particular, AQP1 was strongly expressed in centroacinar cells and both in apical and basolateral domains of intercalated and intralobular duct epithelia. Moreover, AQP5 was observed in the apical membrane of intercalated duct cells and in the duct-associated mucoid glands [23]. Differently, AQP8 immunoreactivity was shown in the apical plasma membrane domains of human acinar cells near the zymogen granules [23], confirming previous data reported in rats [33]. Immunohistochemical analysis revealed AQP12 staining at the basal side of the intracellular organelles of acinar cells close to the nucleus but not in either the duct cells or the islet cells [40].

The presence and specific distribution of AQPs in gallbladder have been investigated using immunohistochemical analysis and protein analysis demonstrating their localization in cell plasma membranes and intracellular vesicles of the gallbladder epithelium of humans and mice [59,60]. In particular, immunofluorescence and immunohistochemical studies showed strong AQP1 and AQP8 signal at the apical membrane of the mouse gallbladder epithelium [24,34]. Moreover, other data show a similar distribution in the gallbladders of wild-type and AQP1 null mice, with comparable epithelial thickness and cell density [24].

## 3. Gut AQP Function

The function of the digestive system is a complex of metabolic reactions involving the small and large intestine as well as the liver, pancreas, and gallbladder. Indeed, along the intestinal wall, the presence of different specialized cells and that of gut microbiota allow multiple processes to be set in train that terminate with the production of feces. Finally, gut function comprises not only nutrient absorption and secretion, but also processes such as homeostasis, regulation of resistance to disease, and production of factors involved in cell growth and repair [16]. In recent decades, the presence of different AQPs distributed through the gut has been convincingly demonstrated. These studies conducted in humans and rodents highlighted an interesting aspect regarding their possible involvement in different gut processes, namely participation in the plasticity and adaptability of the gut in relation to diet. In normal conditions in a human, about 1.5–2 L of water is absorbed daily by the colon, while the maximal capacity of the intestine to absorb fluids may be as high as 5–6 L per day [61]. The exact mechanism by which fluids are transported in the epithelia of the gastrointestinal tract has largely focused on whether water passes through cells (transcellular) or between cells (paracellular). According to the majority of the studies, the transport at the level of gastrointestinal tract is in most cases transcellular [62], even if it is still believed that absorption of water in the small intestine occurs primarily in a paracellular manner [63] or by cotransporters. The importance of the paracellular passage is also supported by research data obtained in AQP5 knockout mice, reporting that in these animals the decrease in transcellular water transport in parotid glands can be associated with an increase of paracellular permeability to ions [64,65]. A significant decrease in tight junction proteins, claudin-7, and occludin was observed in the AQP5^−/−^ knockout mice, even if the molecular pathways remain elusive, these studies indicated that AQP5 could function to link paracellular and transcellular pathways. On the other hand, the hypothesis of water transport via co-transporters has been demonstrated for water uptake in intestinal epithelial cells which, as reported by the authors, have a very low expression of AQPs [66].

In the duodenum, water is secreted and its transport is affected by gastrointestinal hormones (i.e., gastrin, vasoactive intestinal peptide (VIP), and others), neurotransmitters, and histamine, which contribute to water and electrolyte balance [30]. Most of the intestinal water is absorbed in an isosmotic fashion by the small intestine and only in part by the large intestine, according to their different electrical resistance characteristics—as demonstrated for AQP7, which is involved in the physiological mechanisms of fluid absorption and secretion [54]. The discovery of a specific water channel and highly conserved AQPs on epithelial cells in the gastrointestinal tract have identified their role in rapid water movements. However, it is important to consider the cell structure and function along the different tracts in order to examine the functional activity of the different AQPs. According to their transepithelial electrical resistance characteristics, the epithelia from different portions of the intestinal tract may be classified into three categories: leaky (i.e., small intestine), moderately tight (i.e., colon and gastric antrum), and tight (i.e., gastric fundus) [67]. In particular, AQP1 has been demonstrated to be associated with AQP5 (in the duodenum, as well as in the pancreas) [23,30], with an activity probably related to water secretion [30].

Characterization of the columnar absorptive cells in the distal colon demonstrated that these cells express the three subunits of the epithelial Na^+^ channel in the apical membrane [68] and Na^+^-K^+^-ATPase in the basolateral membrane in a similar way to what occurs in the kidney [69]. The renin angiotensin aldosterone system plays a pivotal role in the regulation of water and sodium reabsorption in the kidneys. In particular, vasopressin plays a hormonal function in the mechanism of water homeostasis acting through AQPs. In particular, AQP2 is regulated by antidiuretic hormone (ADH), when plasma osmolarity increases thanks to the endings of magnocellular neurons in the posterior pituitary [69].

AQP1 is expressed in dietary fat processing [70], including cholangiocytes in the liver regulating bile production and pancreatic microvascular endothelium, where it plays a role in pancreatic fluid production [71]. Its activity has been demonstrated also in bile and pancreatic juice, thanks to the evidence that malabsorption problems were observed in AQP1 null-mice [70].

By contrast, AQP3 activity, well known in both the oral cavity and stomach, has also been evidenced in the distal colon: it is expressed and localized along the epithelial cells, at the level of lumen and crypts, suggesting its importance in water transport to the cells involved in the formation of intestinal contents and feces [52]. This data has also been confirmed by studies using HgCl_2_ as an AQP3 inhibitor, used to investigate the role of AQP3 in the regulation of fecal water content [72].

A proliferative activity for this AQP has been demonstrated in enterocytes, evidencing its possible therapeutic use in Crohn’s disease [7]. The role of AQP3 would appear to be related to transcellular water reabsorption, i.e., water transfer from the lumen to the interstitium according to the osmotic gradient [73,74]. AQP7 and AQP8, expressed along the large intestine, seem to play a role in water trafficking from lumen to the interstitium by a transcellular route [32,44]. It is particularly evident for AQP8, confirmed also by higher protein content in intracellular vesicles [45].

Functional studies have demonstrated that both the AQPs’ distribution patterns and expression levels could be modulated by feeding conditions, food preference, and developmental regulation. In particular, a correlation between protein expression and feeding was only observed for AQP6, showing that this protein is upregulated by feeding [75]. In another study conducted in European sea bass (*Dicentrarchus labrax*), short- and long-term fasting influenced metabolic activities and liver AQP9 expression, suggesting that nutritional status could modulate AQP’s role in hepatic glycerol uptake [76]. In addition, in our previous study conducted on buffalo calves fed with colostrum for one week, a different pattern of AQP1, 4, and 5 expression was observed with respect to the control group fed milk. These data indicate that a specific regulation of AQP function and distribution could be achieved according to the nutritional level or the food preference [77,78,79].

## 4. Liver AQP Function

The liver is a well-known metabolic organ sensible to nutrients and hormones. The involvement of AQPs in the liver must also be considered on the basis of their identification and different distribution in several hepatic cell types, as recently demonstrated [22,41]. The cited authors have charted in the human liver and in both human and mouse hepatocytes the presence of several AQPs providing interesting gene regulation by known drugs/hormones (i.e., dexamethasone, forskolin, rosiglitazone, and ghrelin). Recently, Laforenza et al. [41] have clarified the relationship between liver aquaglyceroporin expression and adipose regulation. The authors evidence the important AQP role in physiological conditions, in obesity and type 2 diabetes, identifying these proteins as potential therapeutic targets for metabolic disorders. The AQP9 hepatic role has been extensively studied, confirming its involvement in glycerol metabolism as well as in gluconeogenesis [80,81,82]. In particular, some studies have also clarified the role of protein deletion or protein functional modification using genetic model or specific inhibitors. For example, in livers of female AQP9^+/+^ mice, the immunohistochemical protein signal was more intense in hepatocytes close to the central vein (perivenous hepatocytes), whereas in male mice the label appeared to be more uniformly distributed in all the hepatocytes. As expected, AQP9^−/−^ mice did not show any immunostaining [83]. In the meantime, studies on primary hepatocyte culture treated with a specific novel AQP inhibitor, HTS13286, revealed the metabolic importance of t AQP9, which plays a role in the control of glycerol uptake after hyperglycemia induction [84]. However, additional studies will be required to understand the transcriptional regulation of AQPs in the liver under pathophysiological conditions.

**Figure 2:**
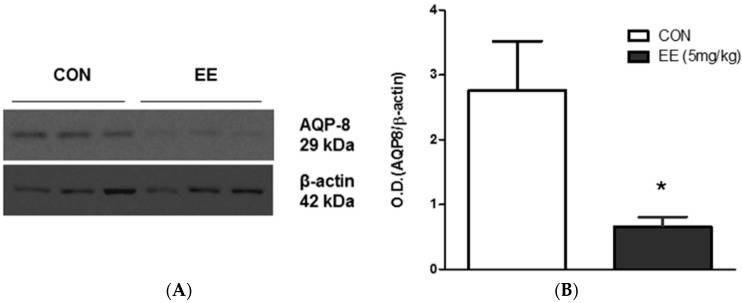
(**A**) AQP8 protein expression in the liver of untreated (CON, control animals receiving propylene glycol as drug vehicle, s.c.) and cholestatic mice (EE). Cholestasis was induced by ethinyl estradiol (5 mg/kg/die for five days, s.c.). Male 10-week-old BALB/c mice were killed at day 5, 1 h after drug treatment; (**B**) Representative image of densitometric analysis of AQP8 protein band (28 kDa) is shown (*n* = 6). All data are expressed as mean ± SEM. Equal loading was confirmed by β-actin staining. Statistical analysis was performed by Student’s *t*-test analysis. * *p* < 0.05 vs. control group.

Recently, new data and knowledge regarding AQP involvement in bile formation as well as in the development of bile secretory failure were reviewed [85]. During active choleresis, hepatocytes increase vesicle trafficking and bile passage, enhancing canalicular membrane water permeability. AQP8 modulates membrane water permeability, providing a molecular mechanism for the osmotically-coupled transport of solute and water during bile formation. Cholestasis is a pathologic condition defined as an impairment of normal bile formation, which may or may not be associated with bile flow obstruction. Estrogens are known to cause intrahepatic cholestasis in women during pregnancy and many drugs, such as oral contraceptives or postmenopausal replacement therapy, can induce this disorder [85]. A murine model of cholestasis, induced by 17α-ethinylestradiol (EE), has been used to investigate alterations in the expression of hepatocyte membrane transporters in this pathology. Our data reported in Figure 2A,B show a clear reduction in AQP8 expression in this experimental model.

Generally speaking, in the liver, as in the other tissues, the regulation in AQP activity must take into account their structure at the C-terminal level for the interaction with other proteins, as well as the Ca^2+^-binding sites, an N-terminal conformational switch, and trafficking in the inner molecule. It must be added that some AQPs require an activation mechanism for their permeability regulation [86].

## 5. Pancreas and Gallbladder AQP Functions

Different studies have been performed to evaluate the exact functional contribution of AQPs in the pancreas and gallbladder to gain better comprehension of their role in pathological conditions. Based on limited published data, it is possible to confirm that water transport in the pancreas occurs both by paracellular and transcellular pathways [14]. In particular, the evaluation of AQPs’ role as water channels in the osmotic permeability of the acinar cell membrane was examined with the help of an Hg^2+^ inhibitor, which is known to effectively block most of the AQP isoforms, including AQP8 [87]. In particular, the treatment of the pancreas secretory vesicles with this inhibitor caused vesicle swelling, confirming the involvement of AQP1 in rapid gating of water in zymogen granules [88]. In the meantime, studies with knock-out (KO) mice (for AQP1 and AQP8) showed that pancreatic secretion is not significantly affected by AQP1 deletion [70]. Similarly, in another study, AQP12 deletion did not affect the overall pancreatic exocrine function in mice under a normal breeding environment, while AQP12-KO mice showed a more severe pathology resulting from CCK-8 analog-induced pancreatitis than wild type (WT) mice [40]. The confirmation of the role of some AQPs in pancreas exocrine function has been obtained by studies on liver X receptors (LXRs) β^−/−^ mice, where a pancreatic exocrine insufficiency has been associated with a reduction in AQP1 expression [89].

Regarding the gallbladder function, different studies revealed the presence of AQP1 and AQP8, confirming their role as a protein directly involved in water transport across the apical membrane of gallbladder epithelium [24,34]. Moreover, a study on gallbladder AQP1-deficient mice demonstrated a strong reduction of water permeability in these animals, indicating that AQP1 provides the principal route for osmotic water transport by a transcellular rather than a paracellular pathway [24]. Functional gallbladder studies revealed changes of AQP expression and the absorptive function of this organ in mice fed a lithogenic diet, suggesting their involvement in water and electrolyte transport [90].

## 6. AQPs in Gut Pathophysiology

Altered expression of AQPs have been identified as co-factors in the etiopathogenesis of some gastroenteric disorders [91,92]. Diarrhea represents a common pathology and is widely studied using different animal models. It is characterized by two important conditions: (1) transepithelial hypersecretion of fluid in the gastrointestinal (GI) tract and (2) defects in water absorption in the colon. Both are important factors that suggest the definite involvement of AQPs.

In a model of attaching and effacing pathogen-induced diarrhea, an evident alteration in AQP distribution (especially AQP2 and AQP3) has been evidenced in colonocytes [92]. In the same way, AQP4 and AQP8 are shown to decrease significantly in a mouse model of colitis after exposure to dextran sodium sulfate (DSS), confirming data obtained from clinical investigations in inflammatory bowel disease (IBD) patients [91]. Further studies in 2008 [93] and in 2010 [94] clarified that the decrease in AQP expression in enterocyte membranes might be the cause of restricted water re-absorption, leading to diarrhea generation. Modification of the AQP pattern seems to be attributed to a translocation mechanism mediated, at least in part, by EspF and EspGt [95].

Recent studies on inflammatory bowel disease (IBD), including Crohn’s disease (CD) and ulcerative colitis (UC), have suggested a particular role played by AQPs [96]. Aquaporin expression, especially AQP4, 7, and 8, was examined in a murine model of colitis and in patients with IBD or infection colitis [89]. The expression of AQP4 and AQP8 mRNA and protein was slight but significantly decreased, while AQP7 was more variable.

Colitis can be aggravated by stress, like sleep deprivation, and improved by anti-inflammatory agents including melatonin [97]. In microarrays and real-time PCR on the mouse colon, mRNA of adiponectin and AQP8 were downregulated by sleep deprivation and upregulated by melatonin.

Our unpublished data obtained on DSS-induced colitis in mice show a slight but significant reduction of AQP1 expression in cecum tracts (data not shown), determined using an antibody that recognized a specific band at 28 kDa and two other bands at 55 and 65 kDa, respectively. These last two bands, as previously evidenced by other authors in the avian small intestine, could presumably be associated to glycosylated forms [98]. At the same time, pharmacological treatment with sodium butyrate, a short-term fatty acid (SCFA) that is considered a fuel for intestinal epithelial cells, reverted the pathologic conditions, reducing the effect of colitis by normalizing AQP1 expression. As is well recognized, this SCFA modulates different processes in the gastrointestinal tract such as electrolyte and water absorption [99]. These data for the first time confirm the involvement of AQP1 in colitis, suggesting further studies to clarify the mechanisms involved in AQP dysfunction. These findings provide new information regarding the role of these membrane channels in IBD, focusing on their dysregulation in gut permeability and bidirectional water/glycerol transit induced by chronic inflammation.

A role for AQPs was also investigated in celiac disease, another intestinal inflammatory disorder induced in genetically susceptible subjects by gluten ingestion [31]. Studies in this regard have demonstrated a dramatic reduction in both AQP mRNA and protein expression combined with a reduced activity of principal solute transporters in the villus (i.e., SGLT1, PEPT1, and NHE3).

Moreover, in a model of allergic diarrhea, a disease resulting in immunological and microbiological homeostasis alterations, decreased expression of AQP4 and AQP8 was observed [100]. These data provide insights into the intestinal role of AQPs, defining a new mechanism to wash the food allergen out of the gastrointestinal tract. AQPs have also been studied in other diseases where fluid flux alterations may contribute to increased susceptibility to injury in the small intestine (i.e., shock after early ischemic injury) [101], introducing a new concept of the intestinal mucosa barrier.

Accordingly, AQP3 expression alterations could be associated with modifications of transcellular and paracellular water transport causing intestinal and endotoxin translocation [102].

## 7. AQP Expression and Distribution in the Gut of Other Species

### 7.1. Small Intestine

To date, several aquaporin isoforms have been identified in the gut of different animal species. Expression and localization of aquaporin isoforms in the digestive system are summarized in Table 2.

AQP1-immunoreactivity (IR) was found in endothelial cells of capillaries, small vessels, and central lacteals in the villi of the small intestine in the pig, rat, mouse, and buffalo [10,26,60,70,77,103,104]. In addition, AQP1-IR was also found in the enterocytes of the crypts in the buffalo small intestine [77], in the apical and basolateral membranes of Brunner’s gland cells in the rat duodenum [30], and in enteric neurons of the buffalo, rat, and sheep small intestine [77,105,106]. RT-PCR and Northern blot analysis confirmed AQP1 mRNA expression in the small intestine of buffalo, pigs, and rodents [10,77,103]. Moreover, AQP3-IR has been found only in the basolateral membrane of the epithelial cells in the villous tip of the rat small intestine [26,107,108], while in humans AQP3-IR is localized also in the enterocytes, goblet cells, and Paneth cells of the crypts [20,25,31]. AQP3 mRNA expression, using Northern blot, in situ hybridization analysis, and real-time RT-PCR, was found in the rat small intestine [107,108,109], suggesting a specific role for AQP3 as a water output modulator after an absorption process via the transcellular pathway [107], which involves SGLT1 or a combination of other transporters.

AQP4-IR is distributed at the basolateral membrane of the cryptic cells located at the bottom of the crypt of the rat, guinea pig, buffalo, and porcine small intestine [10,78,110,111]. AQP4 was also found in the enterocytes along villi and in the Brunner glands [78,110,111] and revealed by Western blot analysis in the rat, guinea pig, mouse, and buffalo small intestine [78,104,109,110]. AQP4 mRNA expression was confirmed in the rat and buffalo small intestine [78,109].

In the rat duodenum, AQP5-IR is present in the apical and lateral membranes of Brunner’s gland secretory epithelium [19,112] and increases in the apical membranes of these cells after stimulation by vasoactive intestinal peptide [30]. Rat AQP5 mRNA has also been found in the duodenum [19].

The presence of AQP5 in the proximal region of the small intestine suggests its secretory role induced by several mediators [113]. Additionally, a study on the gastrointestinal tract of the chicken demonstrated the presence of ck-AQP5 in the crypt cells of the jejunum, ileum, and rectum, while it was found to be absent in the epithelial cells lining the villi. Abundance of ck-AQP5 mRNA and protein was higher in the jejunum, decreasing towards the colon [114]. In addition, AQP5-IR and mRNA were found in the enterocytes and endocrine cells of the buffalo small intestine [78].

In the rat small intestine, AQP6-IR and AQP7-IR as well as proteins and relative mRNAs were present in the apical part of the epithelial cells of the villus [54,75]. Moreover, AQP8-IR was localized in the subapical site of rat duodenum and proximal jejunum enterocytes [44,45,108,115]. On the contrary, in the human duodenum AQP8 does not seem to be expressed [31] although it was found in the human ileum [96]. Using semi-quantitative and real-time RT-PCR, AQP8 was detected in the rat duodenum, proximal jejunum [115], and ileum [108,109]. AQP9-IR was also localized in the goblet cell basolateral membrane of the rat small intestine [35].

Recently, a study on bovine and ovine duodenum showed that AQP10 gene was a pseudogene in these species [116], confirming a previous study conducted on humans and mice [38]. Pseudogenes are generally produced by gene duplications, which preserve the original function or could acquire a new function to survive in an unsafe environment in the form of pseudogenes. The authors suggest that in the case of bovine AQP10 pseudogene its function could be compensated for by other aquaglyceroporins.

### 7.2. Large Intestine

The AQP1 isoform is localized in the capillary and small vessel endothelium of the rat and buffalo large intestine [26,77] and in the enterocytes of the buffalo [77]. Among birds, AQP1 has been found in the lower intestinal tract of the *Passer domesticus*; in particular, its epithelial distribution was limited to the distal rectum [98].

In the rat colon, AQP2-IR was localized in apical membrane epithelium, and RT-PCR, in situ hybridization, immunoblotting, and immunocytochemistry confirmed its expression in colonic crypts and, to a lesser extent, in surface absorptive epithelial cells [50]. Along the rat large intestine AQP3-IR was evidenced in the distal colon and rectum and localized at the basolateral membrane of the absorptive epithelial cells directly facing the lumen and at the neck of crypts [26]. In addition, in the rat proximal and distal colon, AQP3 protein and mRNA expression were also detected [27,107,108,109].

AQP4-IR was localized on the basolateral membrane of epithelial cells isolated from the rat, buffalo, and porcine colon [53,79,111,119]. In addition, AQP4 expression was found in the endothelium and enteric neurons of the buffalo and rodent colon [79,119]. This localization suggests the involvement of the enteric nervous system in body fluid homeostasis by monitoring changes in osmotic pressure and controlling water movement across the mucosa. The AQP4 protein and relative mRNA were also revealed in the buffalo and rat large intestine [79,109].

AQP5-IR, protein, and relative mRNA were shown in the endocrine cells of the buffalo large intestine [79] while AQP7-IR was distributed in the surface epithelial cells of the crypt of the rat colon and cecum [54,109].

Other data gave additional information about the importance of retrograde peristalsis for water conservation in murine species. In particular, AQP8-IR was localized in the subapical site of the absorptive epithelial cells of the rat colon, suggesting its role in fecal dehydration [43,45,115,123]. AQP8 staining was also observed in the intracellular compartment of the surface epithelial cells of rat proximal colon and rectum [108,115]. Moreover, in rat AQP8 mRNA was detected in the proximal and distal colon, rectum, pancreas, and liver [108,115] and AQP9 was localized at the basolateral membrane of the goblet cells [35].

### 7.3. Liver, Pancreas, and Gallbladder

Some AQPs were also expressed in organs anatomically and physiologically related to the gut. In particular, AQP1, 8, and 9 were evidenced in the liver and pancreas of the rat and pig [26,117]. AQP1 was also localized in the capillary and small vessel endothelium of the liver and pancreas of the rat [26], while AQP9 showed different tissue distribution between rats and pigs. This different tissue distribution could suggest for AQP9 a specific role according to the animal species, as already observed in humans and rat [124]. In the rat, AQP8 was detected in the apical membrane and cytoplasm of hepatocytes [44,45,115,117,120] and in the apical regions of pancreatic acinar cells [33,44]. AQP9 was localized in sinusoidal surface membranes of the rat and pig hepatocytes [45,121,122,125]. As reported in Table 1 and below, there are few studies in human and mice that reported AQP expression and role, while other species are overlooked.

## 8. Conclusions

The detection of AQPs along the gut and their widespread distribution in human and animal species suggest their role in maintaining fluid homeostasis. Recent data on their involvement in the pathologies of digestive tract have further defined their mechanisms of action and possible applications in therapy using specific modulators. In recent years, new knowledge regarding barrier integrity and how different conditions (trauma, shock, etc.) can induce multiple intestinal reactions (intestinal cytokine response, translocation of intestinal bacteria, systemic inflammatory response syndrome) has stimulated research in the field of AQPs. In the meantime, new research approaches based either on genetic studies by the use of knockout mice and functional studies by the use of specific AQPs inhibitors have opened a pathway to new possibilities for clinical therapy. In particular, AQPs inhibitors could be used as probes to assess their function in several disease models, and without the need for RNA silencing or knockout models, which have additional limitations and drawbacks, such as adaptive changes in phenotypes. Pharmacological inhibition of AQP water permeability in epithelia, with consequent reduced fluid transport, was also reviewed [126], suggesting AQP modulation as a potential therapeutic target for human diseases involving water imbalance such as congestive heart failure, hypertension, and glaucoma.

Even if this research area is underdeveloped, two recent reviews by Verkman et al. (2014) [13] and Beitz et al. (2015) [127] provide an overview on AQP-related disorders and pharmacological intervention in the therapeutic modulation of aquaporin functionality, identifying protein structural and chemical aspects of AQP modulator design. Indeed, the modulation of AQP functions is desirable in other several pathophysiological situations not only in the gut, such as cancer, heart failure, nephrogenic diabetes insipidus, and Sjögren’s syndrome.

## Figures and Tables

**Table 1:** Gut distribution, selective permeability, and tissue localization of AQPs in human and murine tissues.

AQP Isoform	Selective Permeability	Gut Distribution								Cellular Localization	References
		Small Intestine			Large Intestine		Liver	Pancreas	Gallbladder		
		Duodenum	Jejunum	Ileum	Caecum	Colon					
AQP1	H_2_O	+	n.d.	+	+	+	+	+	+	capillary endothelium of the mucosa and submucosa (duodenum, ileum), crypt epithelium (colon), acinar cells (pancreas), apical and intercellular membrane epithelium (gallbladder)	Matsuzaki, T., 2003 [19]; Mobasheri, A., 2004 [20]; Laforenza, U., 2012 [21]; Gregoire, F., 2015 [22]; Burghardt. B, 2003, [23]; Li, L., 2009 [24]
AQP2	H_2_O	+	+	+	n.d.	n.d.	n.d.	n.d.	n.d.	mucosa	Mobasheri, A., 2005 [25]
AQP3	H_2_O, glycerol, urea	+	+	+	n.d.	+	+	n.d.	n.d.	basolateral membrane of epithelial cells at luminal surface (intestine), Kupffer cells, and hepatocytes	Matsuzaki, T., 2004 [26]; Ishibashi, K., 1995 [27]; Silberstein, C., 1999 [28]; Ikarashi, N., 2013 [29]; Mobasheri, A., 2005 [25]; Laforenza, U., 2012 [21]; Gregoire, F., 2015 [22]
AQP4	H_2_O	+	n.d.	n.d.	n.d.	+	n.d.	n.d.	n.d.	Basolateral membrane of crypt cells and of epithelial cells at luminal surface	Matsuzaki, T., 2004 [26]
AQP5	H_2_O	+	n.d.	n.d.	n.d.	n.d.	n.d.	+	n.d.	apical membrane of duodenal gland secretory cells, intercalated duct cells (pancreas)	Matsuzaki, T., 2004 [26]; Parvin, M.N., 2005 [30]; Burghardt. B, 2003 [23]
AQP7	H_2_O, glycerol, urea	+	+	+	n.d.	+	n.d.	n.d.	n.d.	Superficial epithelial cells	Laforenza, U., 2010, 2012 [21,31]; Gregoire, F., 2015 [22]
AQP8	H_2_O	n.d.	n.d.	n.d.	+	+	+	+	+	subapical site of absorptive epithelial cells, hepatocytes and cholangiocytes, apical region of acinar cells (pancreas), apical membrane epithelium (gallbladder)	Laforenza, U., 2010, 2012 [21,31]; Matsuzaki, T., 2004 [26]; Fisher, H., 2001 [32]; Koyama, Y., 1999 [10]; Gregoire, F., 2015 [22]; Burghardt. B, 2003 [23]; Hurley, P.T., 2001 [33]; Ambe, P.C., 2016 [34]
AQP9	H_2_O, glycerol, urea	+	n.d.	+	n.d.	n.d.	+	n.d.	n.d.	basolateral membrane of goblet cells, cholangiocytes	Matsuzaki, T., 2004 [26]; Okada, S., 2003 [35]; Gregoire, F., 2015 [22]; Mobasheri, A., 2004 [36]
AQP10	H_2_O, glycerol, urea	+	+	+	n.d.	n.d.	n.d.	n.d.	n.d.	enterocytes, gastroenterohepatic (GEP) endocrine cells, and pseudogenes in mice	Li, H., 2005 [37]; Morinaga, T., 2002 [38]
AQP11	Unknown	+	+	+	n.d.	+	+	n.d.	n.d.	enterocytes, hepatocytes	Laforenza, U., 2010, 2012 [21,31]; Ishibashi, K., 2014 [39]
AQP12	Unknown	n.d.	n.d.	n.d.	n.d.	n.d.	n.d.	+	n.d.	acinar cells (pancreas)	Ohta, E., 2009 [40]

n.d. = not determined; + presence.

**Table 2:** Gut distribution of AQPs in different animal species (n.d. = not determined; + presence).

AQP Isoform	Animal Species	Gut Distribution			Cellular Localization	References
		Small Intestine	Large Intestine	Liver		
AQP1	Rat	+	+	+	endothelium of capillaries, small vessels and lacteals of the villi; apical and basolateral membrane of cells of Brunner’s gland ; enteric neurons; hepatic sinusoids	Nielsen, S., 1993 [60]; Koyama, Y., 1999 [10]; Matsuzaki, T., 2004 [26]; Parvin, M.N., 2005 [30]; Nagahama, M., 2006 [105]; Talbot, N.C., 2003 [117]
	Mouse	+	n.d.	n.d.	endothelium of capillaries and small vessels	Ma, T., 2001 [70]
	Buffalo	+	+	n.d.	endothelium of capillaries and small vessels; enterocytes of the crypts; enteric neurons	De Luca, A., 2015 [77]
	Pig	+	n.d.	+	endothelium of lacteals of the villi, liver bile duct	Jin, S.Y., 2006 [103]; Talbot, N.C., 2003 [117]
	Sheep	+	n.d.	n.d.	enteric neurons	Arciszewski, M.B., 2011 [106]
	Birds (*Passer domesticus*)	n.d.	+	n.d.	epithelial cells of the distal rectum	Casotti, G., 2007 [98]
AQP2	Rat	n.d.	+	n.d.	apical membrane of surface columnar epithelial cells	Gallardo, P., 2001 [50]
AQP3	Rat	+	+	n.d.	basolateral membrane of the epithelial cells in the villous tip	Matsuzaki, T., 1999,2004 [26,52]; Ishibashi, K., 1995 [27]; Ramirez-Lorca, R., 1999 [107]; Zhao, G.X., 2016 [108]
AQP4	Rat	+	+	n.d.	basolateral membrane of the cryptic cells and surface of colon epithelial cells; enteric neurons of the colon	Koyama, Y., 1999 [10]; Frigeri, A., 1995 [118]; Wang, K.S., 2000 [53]; Thi, M.M., 2008 [119]
	Mouse	+	+	n.d.	ileal and colon mucosa	Cao, M., 2014 [104]
	Buffalo	+	+	n.d.	enterocytes of the crypts; endothelium	Squillacioti, C., 2015 [78]; Pelagalli, A., 2015 [79]
	Pig	+	+	n.d.	enterocytes along the villi and in the bottom of the crypts and in the Brunner’s gland	Arciszewski, M.B., 2015 [111]
	Guinea pig	+	n.d.	n.d.	enterocytes of the crypts	Jiang, L., 2014 [110]
AQP5	Rat	+	n.d.	n.d.	apical and lateral membranes of the secretory cells of Brunner’s gland	Parvin, M.N., 2005 [30]; Matsuzaki, T., 2004 [26]
	Chicken	+	+	n.d.	enterocytes of the crypts	Ramirez-Lorca, R., 2006 [114]
	Buffalo	+	+	n.d.	enterocytes of the crypts; endocrine cells	Squillacioti, C., 2015 [78]; Pelagalli, A., 2015 [79]
AQP6	Rat	+	n.d.	n.d.	apical region of the enterocytes in the villi	Laforenza, U., 2009 [75]
AQP7	Rat	+	+	n.d.	apical region of the enterocytes in the villi; epithelial cells of the colon and caecum	Laforenza, U., 2005 [54]
AQP8	Rat	+	+	+	apical region of the enterocytes in the villi and of the epithelial cells of the colon; hepatocytes	Calamita, G., 2001 [115]; Elkejer, M.L., 2001 [45]; Tani, T., 2001 [44]; Garcia, F., 2001 [120]; Huebert, R.C., 2002 [57]
AQP9	Rat	+	+	+	basolateral membrane of the goblet cells, hepatocytes	Okada, S., 2003 [35]; Talbot, N.C., 2003 [117]; Nicchia, G.P., 2001 [121]; Huebert, R.C., 2002 [57]
	Pig	n.d.	n.d.	+	hepatocytes	Talbot, N.C., 2003 [117]; Caperna, T.J., 2007 [122]
